# Coronavirus Disease 2019-Related Multisystem Inflammatory Syndrome in Children: A Systematic Review and Meta-Analysis

**DOI:** 10.1155/2021/5596727

**Published:** 2021-07-15

**Authors:** Ji-Gan Wang, Zhi-Juan Zhong, Meng Li, Jun Fu, Yu-Heng Su, You-Min Ping, Zi-Ji Xu, Hao Li, Yan-Hao Chen, Yu-Li Huang

**Affiliations:** ^1^Pediatrics Department, Maternal and Child Health Hospital of Guangxi Zhuang Autonomous Region, Nanning, China; ^2^Pediatrics Department, Hainan General Hospital, Hainan Affiliated Hospital of Hainan Medical University, Haikou, China

## Abstract

**Background:**

This study aimed to describe the clinical symptoms, laboratory findings, treatment, and outcomes of coronavirus disease 2019-related multisystem inflammatory syndrome in children to provide a reference for clinical practice.

**Methods:**

We employed a literature search of databases such as PubMed, Web of Science, EMBASE, and Johns Hopkins University for articles on COVID-19-related multisystem inflammatory syndrome in children published between April 1, 2020, and January 15, 2021. High-quality articles were selected for analysis on the basis of their quality standard scores. Using R3.6.3 software, meta-analyses of random- or fixed-effects models were used to determine the prevalence of comorbidities. Subgroup analysis was also performed to determine heterogeneity.

**Results:**

A total of 57 articles (2,290 pediatric patients) were included in the study. *Clinical Manifestations*. :ncidences of fever, gastrointestinal symptoms, respiratory symptoms, and musculoskeletal symptoms (myalgias or arthralgias) were 99.91% (95% CI: 99.67–100%), 82.72% (95% CI: 78.19–86.81%), 53.02% (45.28–60.68%), and 14.16% (95% CI: 8.4–21.12%), respectively. The incidences of rash, conjunctival injection, lymphadenopathy, dry cracked lips, neurologic symptoms (headache, altered mental status, or confusion), swollen hands and feet, typical Kawasaki disease, and atypical Kawasaki disease were 59.34% (95% CI: 54.73–63.87%), 55.23% (95% CI: 50.22–60.19%), 27.07% (95% CI: 19.87–34.93%), 46.37% (95% CI: 39.97–52.83%), 28.87% (95% CI: 22.76–35.40%), 28.75% (95% CI: 21.46–36.64%), 17.32% (95% CI: 15.44–19.29%), and 36.19% (95% CI: 21.90–51.86%), respectively. The incidences of coronary artery dilation, aneurysm, pericardial effusion, myocarditis, myocardial dysfunction, high troponin, and *N*-terminal pro-B-type natriuretic peptide were 17.83%, 6.85%, 20.97%, 35.97%, 56.32%, 76.34%, and 86.65%, respectively. The incidences of reduced lymphocytes, thrombocytopenia, hypoalbuminemia, elevated C-reactive protein, ferritin, LDH, interleukin-6, PCT, and FIB were 61.51%, 26.42%, 77.92%, 98.5%, 86.79%, 80.59%, 89.30%, 85.10%, and 87.01%, respectively. *PICU Hospitalization Rate and Mortality*. The incidences of PICU hospitalization or with shock were 72.79% and 55.68%, respectively. The mortality rate was 1.00%. *Conclusion and Relevance*. PICU hospitalization and shock rates of multisystem inflammatory syndrome in children associated with COVID-19 were high, and its cumulative multiorgans and inflammatory indicators are increased, but if treated in time, the mortality rate was low.

## 1. Introduction

The transmission of coronavirus disease 2019 (COVID-19) was recognized by the World Health Organization as a global pandemic in March 2020 [1]. In the early stages of the pandemic, it was widely believed that children were not susceptible to infection and the disease was benign disease [2]. However, as the pandemic progressed and children died, people began to pay attention to this part of the population [3]. In April 2020, eight children were reported in the United Kingdom with hyperinflammatory shock, showing features similar to atypical Kawasaki disease—Kawasaki disease shock syndrome [4]. This first report was followed by more in the United States and other regions thereafter [5] and became known as “multisystem inflammatory syndrome in children” (MIS-C). The syndrome was clinically similar to Kawasaki disease (KD), as well as toxic shock syndrome and secondary lymphoid tissue cells hyperplasia/macrophage activation syndrome [6]. Since little was known about this new syndrome, people were extremely worried due to the severity of the condition. Therefore, a systematic description of the clinical manifestations, laboratory tests, and prognosis of this disease is necessary.

## 2. Materials and Methods

### 2.1. Study Registration

The study is reported in accordance with the 2009 guidelines of the Preferred Reporting Items for Systematic Reviews and Meta-Analyses (PRISMA) statement (Supplementary Materials) [7]. This systematic review and meta-analysis is registered in the Prospero International Prospective Register of Systematic Reviews (CRD42020208680).

### 2.2. Definition Criteria

As the disease was discovered in April as a new disease, the name and definition of the disease during the first 2 months were quite different. Therefore, “pediatric inflammatory multisystem syndrome temporally associated with SARS-CoV-2 pandemic (PIMS-TS)” and “multisystem inflammatory syndrome in children (MIS-C)” and “SARS-CoV-2-induced Kawasaki-like hyperinflammatory syndrome (SCiKH syndrome)” were included in the analysis [8].

### 2.3. Computer Retrieval of Articles

Searches for publications reporting on MIS-C associated with COVID-19 in PubMed, Web of Science, Embase, and Johns Hopkins University published data were conducted. The retrieval time for data was from April 1, 2020, to January 15, 2021. At the same time, online database and manual retrievals were conducted, and the references included in the articles were also traced. Subjects and free words were used in the retrieval, and adjustments were made according to the characteristics of different databases. Data retrieval was not limited to any language or country. For the PubMed search strategy, three search categories were used in combination (#1 AND #2 AND #3) as follows:

  #1 (children) OR (child)) OR (kid) OR (pediatric) OR (paediatric)
  #2 (multisystem inflammatory syndrome) OR (MIS-C) OR (PIMS-TS) OR (SCiKH syndrome) OR (SARS-CoV-2–induced Kawasaki-like hyperinflammatory syndrome) OR (Kawasaki)
  #3 (2019-nCoV) OR (COVID-19) OR (SARS-CoV-2) OR (Corona Virus Disease 2019) OR (coronavirus)

### 2.4. Literature Screening and Data Extraction

Two researchers (J. G. W. and Z. J. Z.) independently searched and screened articles as well as collected and cross-checked data. If there was any dispute, it was discussed or negotiated with a third researcher (M. L.).

Inclusion criteria were (1) study type: cohort studies, case-control studies, case series, and cross-sectional studies; (2) participants: (<21 years old) with MIS-C; and (3) observation index: clinical manifestations (including fever and cough), laboratory examination, severe cases, and deaths.

Exclusion criteria were (1) adult cases; (2) case reports; and (3) incomplete, missing, or inaccessible data.

The level of laboratory test items was determined according to the following standards (reference ranges were obtained from Nelson Textbook of Pediatrics): normal white blood cell: 5.5 to 12.0 × 109/L, leukocytosis: more than 12.0 × 109/L, leukopenia: less than 5.5 × 109/L, lymphopenia: less than 1.2 × 109/L, high PCT: more than 0.046 ng/mL, high CRP: more than 10 mg/L, high LDH: more than 300 U/L, high ALT: more than 45 U/L, high AST: more than 50 U/L, high creatinine: more than 62 *μ*mol/L, high blood urea nitrogen: more than 7.1 mmol/L, high CK: more than 170 U/L, high CK‐MB: more than 25 U/L, high D‐dimer: more than 0.55 mg/L, high ferritin: more than 150 ng/mL, high interleukin-6: more than 5 pg/mL, and high *N*-terminal pro-B-type natriuretic peptide: more than 194.0 pg/ml. We excluded studies that did not report original data or clear diagnostic criteria and no relevant outcome.

Diagnostic criteria of coronary artery dilatation (CAD) and coronary artery aneurysms (CAA) were as follows: *Z* value < 2 is no CAD, 2 ≤ Z value < 2.5 is CAD, 2.5 ≤ *Z* value < 5 is small CAA, CAA inner diameter < 0.8 cm and 5 ≤ *Z* value < 10 is medium CAA, and CAA inner diameter ≥ 0.8 cm and *Z* value ≥ 10 is large or huge CAA.

### 2.5. Quality Evaluation of the Included Studies

This was a case series study that adhered to the National Institute for Clinical excellence guidelines for quality evaluation [9]. The evaluation items were as follows: (1) cases in the case series came from medical institutions at different levels and from different research centers; (2) the research hypothesis or purpose was clearly described; (3) clear reports were included in the exclusion criteria; (4) measurement results were clearly defined; (5) collected data achieved the expected purpose; (6) the patient recruitment period was clearly defined; (7) the main findings were clearly described; (8) the results were analyzed and reported in layers. One point was awarded for each item (maximum 8 points), with a total score ≥ 4 indicating high-quality research. Two researchers independently evaluated the quality and cross-checked the results.

### 2.6. Statistical Analysis

Statistical analyses were conducted using the Meta 4.11-0 Package in R Software Version 3.6.3. Before meta-analysis, the conversion of the original rate to conform to a normal distribution was carried out first. The conversion methods included PRAW (original rate without conversion), PLN (logarithmic conversion), PLOGIT (logit conversion), PAS (arcsine conversion), and PFT (Freeman–Tukey dual arcsine conversion). The meta-analysis was carried out on the normal distribution or mode closest to the state distribution. A random- or fixed-effects model was selected according to heterogeneity: if *P* < 0.1 and *I*^*2*^ ≤ 50%, a fixed model was used; and if *P* < 0.1 and *I*^*2*^ > 50%, the study was considered to have heterogeneity and a random-effects model was used [10]. According to the sample size of each independent study, different weights were given and the effect rate of each independent sample was combined to calculate the incidence rate and 95% confidence interval (CI). To explore heterogeneity, we performed subgroup analyses based on the location (region) and sample size (<50, ≥50). Finally, a funnel graph was created and the publication offset was statistically tested using the Egger method.

### 2.7. Ethics

As this is a systematic review, ethical approval was not required.

## 3. Results

### 3.1. Literature Screening Process and Results

**Figure 1:**
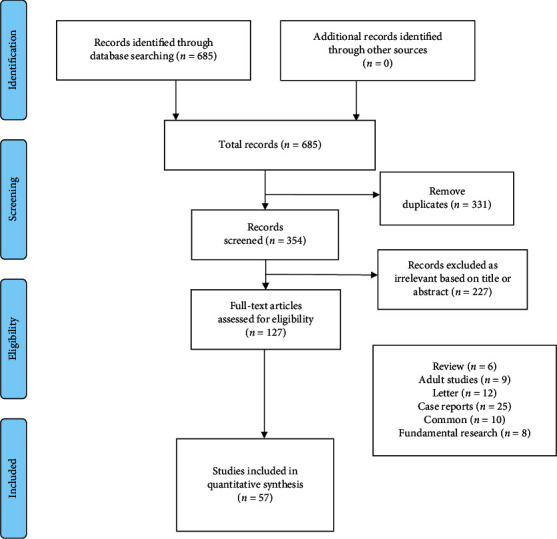
Flow diagram for identification of selected studies in the meta-analysis.

A total of 685 related articles were initially retrieved, of these, 331 duplicate articles were deleted and an additional 227 articles were deleted based on a review of the titles and abstract. Finally, after layer-by-layer screening, a total of 57 articles [5, 11–66] were included (2,290 children), the majority of which were from Europe and the United States. The male to female ratio was 1.49 : 1. The literature screening process and results are shown in Figure 1. The characteristics of the included studies are shown in Supplementary Table 1.

### 3.2. Basic Characteristics and Quality Evaluation Results of the Included Studies

The quality characteristics of all included studies were 4–8 points, indicating high-quality studies (≥4 points; Supplementary Table 2).

### 3.3. Meta-Analysis Results (Table 1)

#### 3.3.1. Clinical Manifestations

The incidences of fever, gastrointestinal symptoms, and musculoskeletal symptoms (myalgias or arthralgias) were 99.91% (95% CI: 99.67–100%), 82.72% (95% CI: 78.19–86.81%), and 14.16% (95% CI: 8.4–21.12%), respectively.

The incidences of rash, conjunctival injection, lymphadenopathy, dry cracked lips, neurologic symptoms (headache, altered mental status, or confusion), swollen hands and feet, typical KD, and atypical KD were 59.34% (95% CI: 54.73–63.87%), 55.23% (95% CI: 50.22–60.19%), 27.07% (95% CI: 19.87–34.93%), 46.37% (95% CI: 39.97–52.83%), 28.87% (95% CI: 22.76–35.40%), 28.75% (95% CI: 21.46–36.64%), 17.32% (95% CI: 15.44–19.29%), and 36.19% (21.90–51.86%), respectively.

#### 3.3.2. Cardiac Damage

The incidences of coronary artery dilation, aneurysm, pericardial effusion, myocarditis, cardiac systolic function affects, high troponin, and *N*-terminal pro-B-type natriuretic peptide were 18.2% (95% CI: 12.0–25.5%), 6.7% (95% CI: 2.8–12.1%), 21.5% (95% CI:14.3–29.7%), 36% (95% CI: 32.9–39.1%), 62.5% (95% CI: 51.2–73.3%), 79.6% (95% CI: 66.7–90.0%), and 89.7% (95% CI: 79.1–96.7%), respectively.

#### 3.3.3. Laboratory Examinations

The incidences of reduced lymphocytes, thrombocytopenia, hypoalbuminemia, elevated white blood cell count, leukopenia, C-reactive protein (CRP), ferritin, LDH, erythrocyte sedimentation rate (ESR), interleukin-6 (IL-6), PCT, and FIB were 61.51% (95% CI: 50.73–71.74%), 26.42% (95% CI: 18.19–35.58%), 77.92% (95% CI: 66.00–87.85%), 55.30% (95% CI: 40.47–69.66%), 4.40% (95% CI: 0.14–14.13%), 98.5% (95% CI: 95.04–99.65%), 90% (95% CI: 80.0–97.0%), 80.59% (95% CI: 42.53–99.73%), 82.44% (95% CI: 73.47–89.89%), 89.3% (95% CI: 75.3–97.88%), 85.10% (95% CI: 75.65–92.55%), and 87.01% (95% CI: 73.97–95.98%), respectively.

#### 3.3.4. Organ Injury

The incidences of liver function damage, kidney damage, and lung ground-glass changes were 46.29% (95% CI: 32.78–60.08%), 27.67% (95% CI: 20.83–35.08%), and 24.69% (95% CI: 19.74–29.99%), respectively.

#### 3.3.5. PICU Hospitalization Rate and Mortality

The incidences of PICU hospitalization or with shock were 72.79% (95% CI: 66.75–78.44%) and 55.68% (95% CI: 48.50–62.74%), respectively. The mortality rate was 1.00% (95% CI: 0.61–1.48%).

#### 3.3.6. Treatment

The proportions of cases using invasive mechanical ventilation, ECMO, IVIG, aspirin, glucocorticoid, vasoactive agent, positive inotropic, IL-1 receptor antagonist, IL-6 receptor antagonist, or infliximab antibiotic were 22.68% (95% CI: 16.91–29.02%), 0.48% (95% CI: 0.03–1.42%), 82.15% (95% CI: 76.53–87.14%), 67.97% (95% CI: 53.77–80.64%), 59.32% (95% CI: 52.02–66.43%), 45.79% (95% CI: 42.62–48.97%), 58.99% (95% CI:48.57–69.02%), 3.61% (95% CI:0.68–8.73%), 10.9% (95% CI: 2.79–23.42%), 6.68% (95% CI: 3.03–11.62%), and 90.96% (95% CI: 82.29–96.92%), respectively.

### 3.4. Subgroup Analysis

#### 3.4.1. Heterogeneity

In this study, except for fever, other factors had obvious heterogeneity (*I*^2^, 39–98). In order to explore the source of heterogeneity, the subjects were classified according to region (United States, Europe, and other countries) and sample size (<50, ≥50) and grouped by gastrointestinal symptoms, lymphopenia, and respiratory symptoms. The results of subgroup analyses were consistent with the overall results, and there was no significant difference between the heterogeneity of each subgroup and overall heterogeneity, indicating that the region and sample size of the study were not the main source of heterogeneity (Table 2).

#### 3.4.2. Sensitivity Analysis

**Figure 2:**
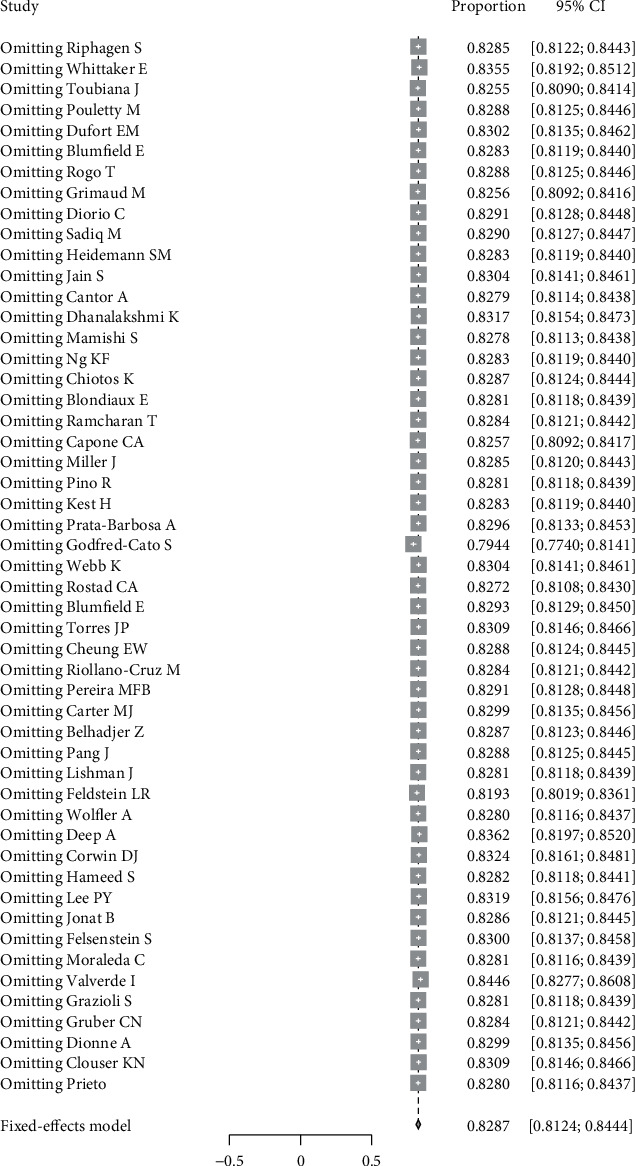
Sensitivity analysis of gastrointestinal symptoms.

Sensitivity analysis was conducted for gastrointestinal symptom indicators. After removing each study in turn, the statistics were combined again. The results did not change significantly, indicating that the results were relatively stable (Figure 2).

### 3.5. Publication Bias

**Figure 3:**
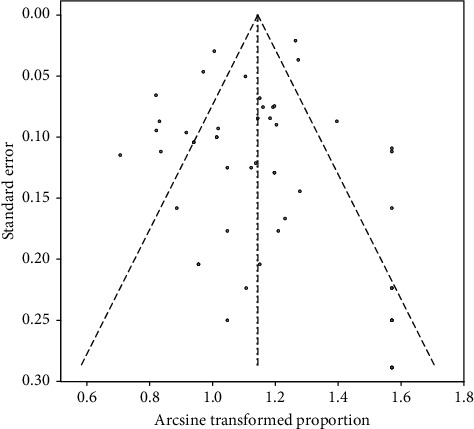
Funnel plot of publication bias of gastrointestinal symptoms.

According to the meta-analysis of gastrointestinal symptoms, a funnel plot was drawn. The results showed that there was good symmetry between the left and right distributions of each research point (Figure 3). The Egger test (*P*=0.943) results suggested that there are no publication biases.

## 4. Discussion

It seems that the nomenclature for multisystem inflammatory syndrome in children has not been unified, with some countries referring to it as “pediatric inflammatory multisystem syndrome temporally associated with SARS-CoV-2 pandemic (PIMS-TS)” or “SARS-CoV-2-induced KD-like hyperinflammatory syndrome (SCiKH syndrome)”; however, “multisystem inflammatory syndrome in children (MIS-C)” is the most commonly used term [67]. Although the definition of this disease is still inconsistent, its common characteristics include fever, multiple organ damage, and increased inflammatory indicators. Since April 2020, eight cases of children with high inflammatory shock COVID-19 were reported in London, UK [5], which increased attention on the recently recognized syndrome due to its severe effects, disbanding the previous notion that children with COVID-19 presented with a mild disease course. Almost 100% of these patients had fever, and only one study from Iran [24] reported patients that did not have fever (4/45 patients). In addition to fever, gastrointestinal symptoms (82.72%) are the second most common clinical manifestation. Because children are often accompanied by cardiac inflammation and some common features of KD, the term COVID-19-related MIS-C was created to describe this novel manifestation and establish diagnostic criteria. The clinical manifestations included repeated high fever, rash, conjunctivitis, peripheral edema, and extensive limb pain, accompanied by obvious gastrointestinal symptoms, which made it difficult to perform volume resuscitation. Finally, norepinephrine and milrinone were needed to provide hemodynamic support. Similar to KD or toxic shock syndrome, most children had no obvious respiratory involvement. The incidence of respiratory symptoms in this study was only 53.02%.

In early stages of the pandemic, this disease was considered to be KD caused by COVID-19, because there are many symptoms of KD, such as rash, conjunctival congestion, chapped lips, and lymphadenitis. However, although there are some phenotypic similarities between MIS-C and KD, there are still many differences between the two diseases, such as the age of onset for KD being <5 years old, [68] and the median age in this study was >5 years old. In KD, platelet count generally increased, while thrombocytopenia accounted for 26.42% of MIS-C cases. Gastrointestinal symptoms in KD and the degree of myocardial dysfunction are also rare, while myocarditis, coronary artery dilatation, cardiac systolic function affects, and gastrointestinal symptoms accounted for 35.97%, 17.83%, 56.32%, and 82.72% of cases, respectively, in MIS-C.

A similar systematic review was published in August this year. The gastrointestinal tract of MIS-C patients [69] was similar to that of this study, and the gastrointestinal symptoms were often abdominal pain. When exploratory abdominal surgery was performed under MIS-C, gastrointestinal investigations also showed mesenteric lymphadenitis and serous effusions (ascites) in severe cases, which implied an active inflammatory reaction had occurred in the digestive system [70]. However, it has been proven that angiotensin-converting enzyme 2 is highly expressed in the small intestine, especially in the proximal and distal intestinal epithelial cells, so the small intestine is more vulnerable to SARS-CoV-2 infection [71].

Laboratory examinations of MIS-C showed a significant increase in various inflammatory indicators, such as CRP, ESR, FIB, ferritin, and LDH. It is worth noting that 89.3% of patients had elevated IL-6, which was as sensitive as 98.5% of patients with elevated CRP. However, lymphocyte reduction was noted in 51.51% of patients, which was much higher than in the majority of cases of COVID-19 in children [72].

Generally, MIS-C is a more systemic disease, increasing the likelihood of organ damage or impairments of liver, kidney, and/or heart function (rather than damaging the respiratory tract or facilitating the pneumonia infection process), which can easily lead to hypotension and circulatory failure. The rate of admission to the PICU was 72.79%, and the rate of shock was 55.68%.

Nevertheless, prognosis is good if appropriate treatment measures are taken as early as possible, such as treatment in the intensive care unit, close monitoring, intravenous injection of immunoglobulin (82.15% utilization as used in the main treatment for KD) and corticosteroids (59.32% utilization), and use of biological agents and anticoagulants under the conditions recommended by appropriate specialists. Although the incidence of severe disease is high, 22.68% of the patients needed invasive mechanical ventilation and few patients (0.48%) required extracorporeal membrane oxygenation, only 1.00% of pediatric patients die, so the overall outcomes are good; however, the long-term cardiovascular outcomes have not been determined.

The limitations of this study are as follows: (1) as the syndrome was known by multiple names at the start of the pandemic, the standards of research inclusion vary from country to country, which may lead to inclusion biases; (2) we found there is great heterogeneity among the studies, and there is significant publishing bias among several subgroups; and (3) this study was analyzed during an ongoing pandemic, and many areas affected by COVID-19 have not yet published clinical datasets, which may lead to inaccurate analysis results.

## 5. Conclusions

The incidence of MIS-C, which presents as multiple organ injuries and systemic inflammatory reactions, is low. MIS-C has a high rate of severity and patients are prone to symptoms of shock; however, if it is identified and treated in time, the mortality rate can remain low.

## Figures and Tables

**Table 1:** Meta-analysis results.

Outcome indicators	Number of included studies	Sample size	Heterogeneity			Effect of model	Meta-analysis results
			*P* values	*I* ^2^	*t* ^2^		*R*% (95% CI)
*Clinical features*							
Fever	47	1367	0.86	0	0.71	Fixed	99.94% (99.74%-100%^)^
Rash	46	1953	<0.01	64.30%	0.0118	Random	59.34% (54.73%-63.87%)
Dry cracked lips	30	1634	<0.01	77.10%	0.0181	Random	46.37% (39.97%-52.83%^)^
Conjunctival injection	37	1799	<0.01	65.70%	0.0112	Random	55.23% (50.22%-60.19%^)^
Swollen hands and feet	24	998	<0.01	81.30%	0.0291	Random	28.75% (21.46%-36.64%^)^
Lymphadenopathy	21	1513	<0.01	86.60%	0.0267	Random	27.07% (19.87%-34.93%^)^
Gastrointestinal symptoms	51	2126	<0.01	80.00%	0.0264	Random	82.72% (78.19%-86.81%)
Neurological symptoms	31	1668	<0.01	82.10%	0.0248	Random	28.87% (22.76%-35.40%)
Respiratory symptoms	36	1753	<0.01	86.85%	0.0378	Random	53.02% (45.28%-60.68%)
Arthralgias	9	393	0.03	53.50%	0.0084	Random	14.16% (8.4%-21.12%)
Typical Kawasaki disease	32	1481	<0.01	92.60%	0.0798	Random	17.32% (15.44%-19.29%)
Atypical Kawasaki disease	18	559	<0.01	91.60%	0.0981	Random	36.19% (21.90%-51.86%)

*Cardiac damage*							
Coronary artery dilation	35	927	<0.01	64.20%	0.0186	Random	17.83% (13.29%-22.87%)
Aneurysm	26	734	<0.01	66.40%	0.0188	Random	6.85% (3.68%-10.92%)
Pericardial effusion	24	1244	<0.01	69.30%	0.0137	Random	20.97% (15.69%-26.79%)
Myocarditis	20	930	<0.01	92.10%	0.098	Random	35.97% (32.92%-39.08)
cardiac systolic function affects	35	1308	<0.01	85.70%	0.049	Random	56.32% (47.65%-64.80%)
High troponin	30	1485	<0.01	95.50%	0.1324	Random	76.34% (63.27%-87.27%)
BNP	31	1554	<0.01	94.20%	0.0971	Random	84.65% (74.95%-92.31%)

*Organ damage*							
Liver function damage	16	411	<0.01	82.20%	0.0524	Random	46.29% (32.78%-60.08%)
Renal function	26	1463	<0.01	84.90%	0.0296	Random	27.67% (20.83%-35.08%)

*Laboratory inspection*							
Leukocytosis	12	401	<0.01	73.30%	0.0394	Random	55.30% (40.47%-69.66%)
Leukopenia	10	417	<0.01	82.90%	0.0514	Random	4.40% (0.14%-14.13%)
Reduced lymphocytes	28	1206	<0.01	89.00%	0.0612	Random	61.51% (50.73%-71.74%)
Elevated platelets	11	421	0.05	44.90%	0.0093	Fixed	10.35% (7.62%-13.43%)
Thrombocytopenia	20	1368	<0.01	87.70%	0.0326	Random	26.42% (18.19%-35.58%
CRP rise	34	1087	<0.01	82.20%	0.0397	Random	98.5% (95.04%-99.65%)
Elevated ferritin	28	884	<0.01	84.00%	0.0483	Random	86.79% (79.32%-92.79%)
Elevated ESR	13	475	<0.01	75.90%	0.0253	Random	82.44% (73.47%-89.89%)
Elevated PCT	17	597	<0.01	81.80%	0.0414	Random	85.10% (75.65%-92.55%)
Elevated FIB	13	414	<0.01	86.80%	0.0658	Random	87.01% (73.97%-95.98%)
hypoalbuminemia	14	457	<0.01	82.70%	0.0443	Random	77.92% (66.00%-87.85%)
Elevated LDH	10	478	<0.01	98.00%	0.3994	Random	80.59% (42.53%-99.73%)
Elevated IL-6	11	527	<0.01	91.90%	0.0814	Random	89.3% (75.3%-97.88%)
Pulmonary ground glass change	14	271	<0.01	71.00%	0.0358	Random	24.69% (19.74%-29.99%)

*Severe disease and mortality*							
PICU	48	1949	<0.01	83.40%	0.0346	Random	72.79% (66.75%-78.44%)
Shock	42	1804	<0.01	85.30%	0.0384	Random	55.68% (48.50%-62.74%)
Mortality	45	2010	<0.01	39.00%	0.0039	Fixed	1.00%(0.61%-1.48%)

*Treatment*							
Invasive mechanical ventilation	46	1565	<0.01	85.45%	0.0451	Random	22.68%(16.91%29.02%)
ECMO	37	1078	<0.01	54.90%	0.0109	Random	0.48% (0.03%-1.42%)
IVIG	47	1959	<0.01	86.00%	0.0413	Random	82.15% (76.53%-87.14%)
Aspirin	20	592	<0.01	89.20%	0.0888	Random	67.97% (53.77%-80.64%)
Glucocorticoid	40	1869	<0.01	86.90%	0.0401	Random	59.32% (52.02%-66.43%)
Vasoactive agent	23	955	<0.01	83.90%	0.0493	Random	45.79% (42.62%-48.97%)
Positive inotropic	24	939	<0.01	87.40%	0.0493	Random	58.99% (48.57%-69.02%)
IL-1 receptor antagonist	16	516	<0.01	76.50%	0.0293	Random	3.61% (0.68%-8.73%)
IL-6 receptor antagonist	18	518	<0.01	91.30%	0.1060	Random	10.9% (2.79%-23.42%)
Infliximab	14	538	<0.10	59.80%	0.013	Random	6.68% (3.03%-11.62%)
Antibiotic	19	528	<0.11	81.9	0.0562	Random	90.96% (82.29%-96.92%)

CRP = C-reactive protein; ESR = erythrocyte sedimentation rate; PCT = procalcitonin; FIB = fibrinogen; LDH = lactate dehydrogenase; IL-6 = interleukin-6; BNP = B type natriuretic peptide; ECMO = extracorporeal membrane oxygenation; IVIG = intravenous immunoglobulin.

**Table 2:** Results of subgroup analysis.

Outcome indicators	Number of included studies	Sample size	Heterogeneity			Effect of model	Meta-analysis results
			*P* values	*I* ^2^	*t* ^*2*^ (%)		*R*% (95% CI)
*Gastrointestinal symptoms*							
US	21	1208	<0.01	72.80	0.015	Random	87.77% (85.86%–89.55%)
Europe	21	753	<0.01	79.90	0.0325	Random	76.53% (73.44%–79.48%)
Other countries	9	165	<0.01	61.60	0.0231	Random	70.54% (63.38%–77.23%)
*N* < 50	44	757	<0.03	70.10	0.0345	Random	84.11% (78.68%–88.87%)
*N* ≥ 50	7	1369	<0.04	94.30	0.0243	Random	78.46% (67.80%–87.46%)

*Respiratory symptoms*							
US	17	1128	<0.01	88.60	0.0393	Random	47.68% (36.53%–58.94%)
Europe	12	489	<0.01	84.90	0.0503	Random	53.77% (38.615–68.59%)
Other countries	7	136	<0.01	84.60	0.0354	Random	67.01% (44.77%–85.83%)
N < 50	30	500	<0.01	81.30	0.0667	Random	54.98% (44.15%–65.57%)
*N* ≥ 50	6	1253	<0.01	94.40	0.0236	Random	50.63% (37.88%–63.33%)

*Lymphocytopenia*							
US	13	1206	<0.01	93.08	0.0729	Random	58.70% (41.92%–74.50%)
Europe	7	77	<0.01	70.90	0.0629	Random	84.01% (63.93%–96.84%)
Other countries	8	134	<0.01	40.70	0.0112	Fixed	48.96% (40.55%–57.40%)
*N* < 50	25	351	<0.01	77.40	0.0627	Random	62.05% (50.28%–73.14%)
*N* ≥ 50	3	855	<0.01	98.40	0.0718	Random	58.4% (28.61%–85.23%)

## Data Availability

The data used to support the findings of this study are included within the article.

## Abbreviations

CRP: C-reactive protein
ESR: Erythrocyte sedimentation rate
PCT: Procalcitonin
FIB: Fibrinogen
LDH: Lactate dehydrogenase
IL-6: Interleukin-6
BNP: B-type natriuretic peptide
ECMO: Extracorporeal membrane oxygenation
IVIG: Intravenous immunoglobulin.

## Supplementary Materials

Supplementary Materials
*Supplementary Table 2*. Literature quality score table. *Supplementary Table 1*. Characteristics of studies included in the meta-analysis.
