# Supplementary material for: Coronavirus Disease 2019-Related Multisystem Inflammatory Syndrome in Children: A Systematic Review and Meta-Analysis
Source: Biochem Res Int. 2021 Jul 15;2021:5596727. doi: 10.1155/2021/5596727 (PMC8324361; doi:10.1155/2021/5596727)
Supplement: Supplementary Materials — Supplementary Table 2. Literature quality score table. Supplementary Table 1. Characteristics of studies included in the meta-analysis. [file 5596727.f1.zip › 5596727.f1/Supplementary eTable 1..doc]

Supplementary eTable 1： Characteristics of studies included in the meta-analysis.

| Author | Region | Research time | Total people | Males | Age |
| --- | --- | --- | --- | --- | --- |
| Riphagen S[6] | UK | Mid-April, 2020 | 8 | 5 | 4-14y |
| Whittaker E[11] | 8 hospitals in England | March 23 and May 16, 2020 | 58 | 38 | Median age 9 y (IQR, 5.7-14y) |
| Toubiana J[12] | Necker Hospital for Sick Children in Paris | April 27 to May11 2020 | 21 | 9 | Median age7.9y (IQR,3.7-16.6) y |
| Pouletty M[13] | even different Paris-region hospitals | Since April 2020 | 16 | 8 | Median age 10 y (IQR, 4.7 to 12.5y) |
| Dufort EM[14] | New York State | March 1 to May 10, 2020 | 99 | 53 | N |
| Blumfield E[15] | Division of Pediatric Radiology, Montefiore Medical Center | Feb. 25 to May 1, 2020 | 3 | 2 | The mean age was 5.75y |
| Rogo T[16] | BronxCare Health System, Bronx, NY, USA | April 26 to May 11, 2020 | 4 | 3 | The mean age was 11.25y |
| Grimaud M[17] | Paris, France | April 15 to April 27, 2020 | 20 | 10 | Median 10y（range,2.9–15y） |
| Diorio C[18] | Children's Hospital of Philadelphia, Philadelphia,US | April 3 to May 15, 2020 | 6 | 2 | Median age 6y (IQR5-7y) |
| Sadiq M[19] | Pakistan. | May 15 to June 30, 2020 | 8 | 7 | Median 9.5y(range was 5–15y ) |
| Heidemann SM[20] | Children's Hospital of Michigan, Detroit, MI, USA | Aug 13,2020 | 3 | 2 | The mean age was 6Y |
| Jain S[21] | Mumbai, India | May 1 to July 15, 2020 | 23 | 11 | Median age 7.2y (IQR5.7 – 9.4y) |
| Cantor A[22] | Columbia University Irving Medical Center, New York, NY, USA. | April 18 to May 22, 2020 . | 44 | 24 | 7.3 years (Range 7 months - 20 years) |
| Dhanalakshmi K[23] | Tamil Nadu, India. | May 4 to July 8, 2020 | 19 | 8 | Median age of 6 y (IQR: 13 m - 16 y) |
| Mamishi S[24] | Iran | March 7 to June 23, 2020, | 45 | 24 | Median 7 y (range ,10 m to 17 y) |
| Ng KF[25] | University of Leicester, Leicester, UK. | April to May 2020 | 3 | 2 | The mean age was 15y |
| Chiotos K[26] | Philadelphia, Pennsylvania, USA. | May28,2020 | 6 | 1 | The mean age was 9.1y |
| Blondiaux E[27] | Sorbonne Université, Paris, France | April, 2020 | 4 | 1 | The mean age was 9 y (range 6-12 y) |
| Ramcharan T[28] | Birmingham Women's and Children's NHS Foundation Trust,UK | April 10 to May 9,2020 | 15 | 11 | Median age of 8.8y (IQR, 6.4-11.2) y |
| Capone CA[29] | Northwell Health, New Hyde Park.US | April 17 to May 13, 2020 | 33 | 20 | Median age of 8.6 y(IQR, 5.5-12.6 y) |
| Miller J[30] | Columbia University Irving Medical Center, New York, NY, USA | April 18 to May 22, 2020 | 44 | 28 | 7.3 y (Range 7 m- 20 y) |
| Pino R[31] | Hospital Sant Joan de Déu, Barcelona, Spain. | March 23 to May 14 | 12 | 9 | Median age of 5.3（IQR,2.5–11.2y） |
| Kest H[32] | Joseph's Children's Hospital, Paterson.US | April 4 to May 10, 2020 | 3 | 1 | The mean age was 8 y |
| Prata-Barbosa A[33] | Brazil. | March 1 to May 31, 2020 | 10 | 8 | The mean age was 5.2y (Range,1.5−8.4y) |
| Godfred-Cato S[34] | CDC.US | March 2 to July 18, 2020 | 570 | 316 | The mean age was 8 y(range, 2w–20 y) |
| Webb K[35] | South Africa, | June 4 to July 24, 2020 | 23 | 17 | N |
| Matsubara D[36] | The Children's Hospital of Philadelphia, Philadelphia.US | April 10 to June 7, 2020 | 28 | 14 | Median age11.4 y(IQR8.0 – 13.7y) |
| Rostad CA[37] | Atlanta, GA, USA. | March 17 to May 26, 2020 | 10 | 6 | Median age8.5y (IQR6.5-12) |
| Blumfield E[38] | Boston Children's Hospital and Harvard Medical school,US | April 22 to May 21, 2020 | 16 | 10 | 20 m - 20 y |
| Torres JP[39] | Chile. | May 1to June 24, 2020 | 27 | 14 | Median age 6y (0-14) y |
| Cheung EW[40] | Columbia University Irving Medical Center, New York, NY, USA | April 18 to May 5, 2020 | 17 | 8 | 8 years (range 1.8-16 y) |
| Riollano-Cruz M[41] | Icahn School of Medicine at Mount Sinai.US | April 24 toJune 19, 2020 | 15 | 11 | The mean age was 12y |
| Pereira MFB[42] | Sao Paulo, SP, BR | April 16 to June 21, 2020. | 6 | 5 | The mean age was 7.78y (range 0.01-17.62) |
| de Farias ECF[43] | Belém, Pará (PA), Brazil. | April 15 to June 15, 2020, | 11 | 9 | The median age was 59 m (7 m to 11 y) |
| Carter MJ[44] | Evelina London Children’s Hospital. UK | April 27 to May25, 2020 | 25 | 15 | Median age 12.5y（IQR, 7.7–14.4y） |
| Belhadjer Z[45] | Paris, France. | March 22 to April 30, 2020 | 35 | 18 | The mean age was 10 y (range, 2-16 y) |
| Pang J[46] | London | late March to mid-May 2020 | 5 | 3 | The mean age was 11y |
| Perez-Toledo M[47] | Birmingham | April 28 to May8, 2020 | 8 | 5 | Median age 9 y(range, 7-14) y |
| Lishman J[48] | Cape Town, Western South Africa. | By August 20th | 4 | 2 | The mean age was 11y |
| Feldstein LR[49] | US | March 15 to May 20, 2020 | 186 | 115 | Median age of 8.3y(IQR,3.3-12.5y) |
| Wolfler A[50] | Milan, Italy | March 15 to April 25, 2020 | 5 | 2 | The mean age was 7y |
| Deep A[51] | United Kingdom | March 14 to May 20, 2020. | 116 | 86 | The median age was 11 y (IQR, 7–14 y |
| Antúnez-Montes[52] | 5 Latin American countries. |  | 95 | 55 | The median age was 3.0 y (IQR 0.6-9.0y) |
| Corwin DJ[53] | Children's Hospital of Philadelphia US | March 1 to May 15, 2020 | 33 | 20 | The median age was 10.9 y |
| Hameed S[54] | Evelina London Children's Hospital UK | April to May 2020 | 35 | 27 | The median age was 11 y |
| Lee PY[55] | Boston Children's Hospital,US | March to June 2020 | 28 | 16 | The median age was 9 y(range, 1m to 17 y |
| Jonat B[56] | Columbia University Irving Medical Center.US | March 15 to June 5, 2020 | 54 | 25 | The median age was 7y (range,0.8-20y), |
| Felsenstein S[57] | Alder Hey Children's NHS Foundation Trust Hospital, Liverpool.UK | March and June, 2020 | 29 | 20 | The median age was 6.0 y (IQR, 3.8–9.9y) |
| Moraleda C[58] | Spanish | March 1 to June 1, 2020 | 31 | 18 | The median age was7.6 (IQR,4.5- 11.5) y |
| Valverde I[59] | 55 centers in 17 European countries | February 1 and June 6, 2020 | 286 | 194 | The median age 8.4 y (IQR 3.8-12.4 y) |
| Grazioli S[60] | Geneva Switzerland. | April 11 and April 26, 2020 | 4 | 4 | The median age was 10 y(IQR,10-13Y) |
| Gruber CN[61] | Icahn School of Medicine at Mount Sinai, NY, NY, USA. | late-April and June 2020 | 9 | 4 | The median age was 12 y |
| Dionne A[62] | Boston Children's Hospital,US | March 1 to May 30, 2020 | 25 | 15 | The median age was 9.7y (IQR2.7-15 y) |
| Clouser KN[63] | Hackensack Meridian School of Medicine.US | April 15 to June 20, 2020 | 20 | 11 | The median age was 7.3 y(Range, 0.88-18y) |
| Prieto[64] | Hospital Universitario 12 de Octubre, Madrid, Spain | April 28 to May 11, 2020 | 5 | 3 | The median age was 7 y (IQR,5-12 y). |
| Falah NU[65] | Mayo Hospital, Lahore, Pakistan | April to July 2020 | 10 | 8 | 4 m to 11 y (mean: 6 y) |
| Kelly[66] | Massachusetts General Hospital, Boston,US | May 1 to June 5, 2020 | 12 | 9 | The median age was 3.5 y(range, 2 m to 21 y) |

N =Not mentioned IQR=Interquartile range
