# Supplementary material for: Coronavirus Disease 2019-Related Multisystem Inflammatory Syndrome in Children: A Systematic Review and Meta-Analysis
Source: Biochem Res Int. 2021 Jul 15;2021:5596727. doi: 10.1155/2021/5596727 (PMC8324361; doi:10.1155/2021/5596727)
Supplement: Supplementary Materials — Supplementary Table 2. Literature quality score table. Supplementary Table 1. Characteristics of studies included in the meta-analysis. [file 5596727.f1.zip › 5596727.f1/Supplementary eTable 2..doc]

Supplementary eTable 2:Literature quality score table

| Author | ① | ② | ③ | ④ | ⑤ | ⑥ | ⑦ | ⑧ | Quality score |
| --- | --- | --- | --- | --- | --- | --- | --- | --- | --- |
| Riphagen S[6] |  | √ |  | √ | √ | √ | √ | √ | 6 |
| Whittaker E[11] | √ | √ | √ | √ | √ | √ | √ |  | 7 |
| Toubiana J[12] |  | √ | √ | √ | √ | √ | √ | √ | 7 |
| Pouletty M[13] | √ | √ | √ | √ | √ | √ | √ | √ | 8 |
| Dufort EM[14] |  |  | √ | √ | √ | √ |  | √ | 5 |
| Blumfield E[15] |  | √ | √ | √ |  | √ | √ | √ | 6 |
| Rogo T[16] |  | √ | √ | √ | √ |  |  | √ | 5 |
| Grimaud M[17] | √ | √ | √ | √ | √ | √ | √ |  | 7 |
| Diorio C[18] |  | √ | √ | √ |  | √ | √ | √ | 6 |
| Sadiq M[19] |  | √ | √ | √ | √ | √ | √ | √ | 7 |
| Heidemann SM[20] |  | √ | √ | √ | √ | √ | √ | √ | 7 |
| Jain S[21] | √ | √ | √ |  | √ |  | √ |  | 5 |
| Cantor A[22] |  | √ | √ | √ | √ |  | √ |  | 5 |
| Dhanalakshmi K[23] |  | √ |  | √ | √ |  | √ | √ | 5 |
| Mamishi S[24] | √ | √ |  | √ | √ | √ | √ | √ | 7 |
| Ng KF[25] |  | √ | √ | √ | √ | √ | √ | √ | 7 |
| Chiotos K[26] |  | √ | √ | √ | √ | √ | √ | √ | 7 |
| Blondiaux E[27] |  | √ | √ |  |  | √ | √ |  | 4 |
| Ramcharan T[28] |  | √ |  | √ |  | √ | √ | √ | 5 |
| Capone CA[29] |  |  | √ | √ | √ | √ | √ | √ | 6 |
| Miller J[30] |  | √ |  | √ | √ | √ | √ | √ | 6 |
| Pino R[31] |  | √ | √ | √ |  | √ | √ |  | 5 |
| Kest H[32] |  | √ | √ | √ |  |  | √ | √ | 5 |
| Prata-Barbosa A[33] |  | √ | √ | √ |  | √ | √ |  | 5 |
| Godfred-Cato S[34] | √ | √ | √ | √ | √ | √ | √ |  | 7 |
| Webb K[35] |  | √ | √ | √ |  | √ | √ |  | 5 |
| Matsubara D[36] |  | √ | √ |  | √ | √ |  |  | 4 |
| Rostad CA[37] |  | √ |  | √ | √ |  | √ |  | 4 |
| Blumfield E[38] |  | √ |  | √ |  | √ | √ |  | 4 |
| Torres JP[39] | √ | √ | √ | √ | √ | √ | √ | √ | 8 |
| Cheung EW[40] | √ | √ | √ | √ | √ | √ | √ | √ | 8 |
| Riollano-Cruz M[41] |  | √ | √ | √ |  | √ | √ | √ | 6 |
| Pereira MFB[42] |  | √ | √ | √ | √ | √ | √ |  | 6 |
| de Farias ECF[43] | √ | √ |  | √ | √ | √ | √ | √ | 7 |
| Carter MJ[44] |  | √ | √ | √ | √ | √ | √ |  | 6 |
| Belhadjer Z[45] | √ | √ | √ | √ | √ | √ | √ | √ | 8 |
| Pang J[46] |  | √ | √ |  |  | √ | √ |  | 4 |
| Perez-Toledo M[47] |  | √ | √ |  |  | √ | √ |  | 4 |
| Lishman J[48] |  | √ | √ |  |  | √ | √ |  | 4 |
| Feldstein LR[49] | √ | √ | √ | √ | √ | √ | √ | √ | 8 |
| Wolfler A[50] |  | √ | √ | √ | √ | √ | √ |  | 6 |
| Deep A[51] | √ | √ | √ | √ | √ | √ | √ | √ | 8 |
| Antúnez-Montes[52] | √ | √ | √ | √ | √ | √ | √ | √ | 8 |
| Corwin DJ[53] |  | √ | √ | √ | √ | √ | √ | √ | 7 |
| Hameed S[54] |  | √ | √ | √ | √ | √ | √ | √ | 7 |
| Lee PY[55] |  | √ | √ | √ | √ | √ | √ | √ | 7 |
| Jonat B[56] |  | √ | √ | √ | √ | √ | √ | √ | 7 |
| Felsenstein S[57] |  | √ | √ | √ | √ | √ | √ | √ | 7 |
| Moraleda C[58] | √ | √ | √ | √ | √ | √ | √ | √ | 8 |
| Valverde I[59] | √ | √ | √ | √ | √ | √ | √ | √ | 8 |
| Grazioli S[60] |  | √ | √ | √ | √ | √ | √ | √ | 7 |
| Gruber CN[61] |  | √ | √ | √ | √ | √ | √ |  | 6 |
| Dionne A[62] |  | √ | √ | √ | √ | √ | √ | √ | 7 |
| Clouser KN[63] |  | √ | √ | √ |  | √ | √ |  | 5 |
| Prieto[64] |  | √ | √ |  | √ | √ |  | √ | 5 |
| Falah NU[65] | √ | √ | √ | √ | √ | √ | √ | √ | 8 |
| Kelly[66] |  | √ | √ |  |  |  | √ | √ | 4 |

① cases in the case series came from medical institutions at different levels and from different research centers; ② the research hypothesis or purpose was clearly described; ③ clear reports were included in the exclusion criteria; ④ measurement results were clearly defined; ⑤ collected data achieved the expected purpose; ⑥ the patient recruitment period was clearly defined; ⑦ the main findings were clearly described; and ⑧ results were analyzed and reported in layers. One point was awarded for each item (maximum 8 points), with a total score ≥4 indicating high-quality research
